# DCE-MRI and IVIM-MRI of rabbit Vx2 tumors treated with MR-HIFU-induced mild hyperthermia

**DOI:** 10.1186/s40349-016-0052-0

**Published:** 2016-03-15

**Authors:** Mie K. Lam, Chris Oerlemans, Martijn Froeling, Roel Deckers, Angelique D. Barten-Van Rijbroek, Max A. Viergever, Chrit T. W. Moonen, Clemens Bos, Lambertus W. Bartels

**Affiliations:** Imaging Division, University Medical Center Utrecht, Utrecht, The Netherlands

**Keywords:** Dynamic contrast-enhanced MRI, Intra-voxel incoherent motion MRI, Hyperthermia, MR-HIFU

## Abstract

**Background:**

The purpose of this study is to investigate whether changes could be detected in dynamic contrast-enhanced (DCE) and intra-voxel incoherent motion (IVIM) MR parameters upon MR-guided high-intensity focused ultrasound (MR-HIFU)-induced hyperthermia in a rabbit Vx2 tumor model.

**Methods:**

Five Vx2 tumor-bearing New Zealand white rabbits were treated with hyperthermia using a clinical MR-HIFU system. Data were acquired before and after hyperthermia. For the DCE analysis, the extended Tofts model was used. For the IVIM analysis, a Bayesian approach was used. Maps were reconstructed of the DCE parameters (*K*^trans^, *k*_ep_, and *v*_*p*_) and IVIM parameters (*D*_*t*_, *f*_*p*_, and *D*_*p*_). Individual parameter histograms and two-dimensional cross-correlation histograms were constructed to analyze changes in the parameters after hyperthermia. Changes in median values were tested for statistical significance with the Mann-Whitney *U* test.

**Results:**

The MR temperature measurements confirmed that mild hyperthermia (40 to 42 °C) was successfully achieved in all rabbits. One rabbit died during treatment and was excluded from the analysis. In the remaining four rabbits, an increase in *D*_*t*_ was observed. In three rabbits, an increase in *K*^trans^ was observed, while in the other rabbits, all three DCE parameter values decreased. Mixed changes were seen for *v*_*p*_ and *f*_*p*_.

**Conclusions:**

Changes in DCE and IVIM parameters were detected after hyperthermia and were variable between the rabbits. DCE- and IVIM-MRI may be promising tools to assess tumor responses to hyperthermia. Further research in a larger number of subjects is necessary in order to assess their value for treatment response monitoring.

## Background

While MR-guided high-intensity focused ultrasound (MR-HIFU) has been used for ablative treatments, the technology also shows promise for the induction of local mild hyperthermia. One of the physiological effects is the improvement of tumor oxygenation, which has been reported to increase the effectiveness of radiotherapy [1, 2]. Other physiological effects are changes in blood flow and vascular permeability, which could enhance local drug delivery of chemotherapeutic agents [3, 4]. Information about tumor physiology is valuable since it is an important determinant of treatment outcomes [5, 6]. Physiological responses of tumors to hyperthermia have been extensively investigated in rodent models using invasive measurement methods [7–13]. Tumors are more sensitive to heating and stasis of the blood flow occurs at lower hyperthermic temperatures as compared with normal tissue [7, 8]. Changes in regional blood flow and permeability after hyperthermia were reported to show both inter- and intra-tumoral variations [7, 13]. The underlying mechanisms are complex and depend on several factors, e.g., the chemical microenvironment and tumor architecture [7], which make it difficult to predict tumor responses to hyperthermia. Noninvasive methods to map physiological changes would therefore be useful for investigating tumor responses to hyperthermia [14, 15].

Dynamic contrast-enhanced (DCE) magnetic resonance imaging (MRI) is a method widely used to map quantitative perfusion and permeability parameters [16, 17]. Dynamic *T*_1_-maps are acquired before, during, and after the injection of a paramagnetic contrast agent bolus. Contrast concentration-time curves are derived from the dynamic *T*_1_ maps, and perfusion parameters can be extracted by fitting a physiological model, such as the Tofts model [18, 19]. Many studies have reported on the potential of DCE-MRI as a prediction tool for treatment response of tumors to radiotherapy [20, 21], neo-adjuvant chemotherapy [22–24], and neo-adjuvant chemoradiation [25–27].

Intra-voxel incoherent motion (IVIM) MRI is a method that allows measurements of perfusion-related parameters from diffusion-weighted MR data. The non-Brownian motion of blood flowing through pseudo randomly organized capillary networks is considered as incoherent motion. This generates a “pseudo diffusion” effect and contributes to the diffusion-weighted MR signal. By using a bi-exponential description of the MR signal, parameters related to the vascularity can be extracted [28]. Although these parameters should be interpreted carefully [29, 30], the vascular contribution to measured IVIM parameters has recently been verified in healthy volunteers [31]. Recent studies showed promising results using IVIM for the characterization of various diseases of different organs, for example, cirrhotic liver [32], pancreatic carcinoma [33], locally advanced breast cancer [34], salivary gland tumors [35], brain pathologies [36], and renal tumors [37].

In this study, we investigated the potential of DCE- and IVIM-MRI to detect changes induced by hyperthermia in rabbits with Vx2 tumors, using the extended Tofts DCE-MRI model and a Bayesian approach for IVIM analysis. To this end, a clinical MR-HIFU system was used, which allows of noninvasive local hyperthermia in small animals [38–42].

## Methods

### Animal handling and Vx2 tumors

All experiments were approved by the University Animal Experiments Committee and were performed in agreement with The Netherlands Experiments on Animals Act (1977) and the European Convention guidelines (86/609/EC). Five female New Zealand white rabbits (2.5–3.5 kg, Charles River, France) were housed in pairs and were provided with food and *ad libitum* water. Vx2 tumor pieces were retrieved from donor rabbits and implanted intramuscularly in the left hind limb. The tumors grew to a volume of 10 cm^3^ in about 3 weeks, after which the imaging experiment was performed.

The rabbits were initially anesthetized with a subcutaneous injection of dexmedetomidine (0.125 mg/kg, Dexdomitor, Jansen Pharmaceutica N.V., Beerse, The Netherlands) and ketamine (15 mg/kg, Narketan 10, Vétoquinol S.A., Lure Cedex, France). The tumor-bearing hind limb was shaved, depilated, and covered with ultrasound gel for acoustic coupling. To prevent undesired leg movement during HIFU exposure, a sciatic nerve block was performed (bupivacaine 2 mg/kg). Then, a fluoroptic temperature probe (Luxtron, Santa Clara, CA) was placed in the muscle tissue adjacent to the tumor to allow measurements of the baseline temperature as is used for the relative MR thermometry.

A catheter (Abbocath®-T I.V. Catheter 22 g × 1.25”, Hospira Inc., Lake Forest, IL) was placed in the marginal ear vein and was connected to a Luer-lock 3-way valve, providing two inputs. One input was used for the intravenous maintenance anesthesia (one third of the initial dose per hour), which was provided using a pressure pump system up to 5 h after the initiation of anesthesia. The other input was available for intravenous injection of the MR contrast agent gadobutrol (GadoVist, 0.1 mmol/kg, Gadovist, Bayer Pharma). After the experiment, the rabbits were terminated with an overdose of sodium pentobarbital injected intravenously.

### Experimental setup

**Fig. 1:**
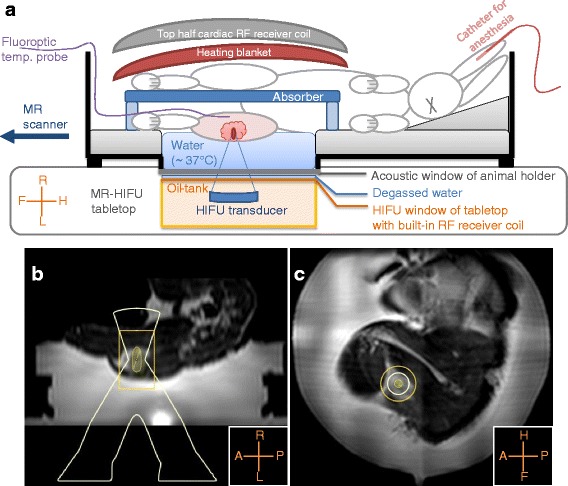
Experimental setup. A schematic overview of the experimental setup is shown in (**a**). The animal holder was placed with its acoustic window above the HIFU window; degassed water was used for acoustic coupling. The shaved tumor-bearing leg was positioned above the acoustic window, and a fluoroptic temperature probe was inserted in the tumor-bearing leg, in the far-field of the HIFU beam. The tank was filled with warm water (~37 °C) up to the tumor-bearing leg, and an absorber was placed between the legs. On top of the rabbit, a heating blanket and a flat 16-channel array coil was placed. In (**b**, **c**), examples of the treatment planning are shown on reconstructed sagittal and coronal images of the T_2_-weighted 3D turbo spin-echo acquisition

A clinical MR-HIFU therapy system was used (Sonalleve V2, Philips Healthcare, Vantaa, Finland) integrated into a clinical 1.5T MRI scanner (Achieva, Philips Healthcare, Best, The Netherlands). An in-house developed animal holder as previously described by Wijlemans et al. [43] was used, which consisted of an open polymethylmethacrylate tank with an acoustic window in the bottom. A schematic overview of the setup is shown in Fig. 1a. The tank was filled with heated water up to the tumor-bearing leg, to enable acoustic coupling and to achieve a baseline temperature similar to human body temperature (37 °C). A heating blanket was placed on top of the rabbit to keep the baseline temperature stable.

### MR imaging

A four-channel RF receiver coil integrated into the MR-HIFU tabletop was used, together with a flat 16-channel array coil, which was placed on top of the heating blanket (Fig. 1a).

To plan the position of the HIFU focus, an anatomical T_2_-weighted 3D turbo spin-echo (TSE) sequence was used with the following scan parameter settings: echo time (TE) = 254 ms, repetition time (TR) = 1000 ms, flip angle (FA) = 90°, TSE factor = 21, acquisition bandwidth (BW) = 245.5 Hz, voxel size = 2 × 2 × 2 mm^3^, field of view (FOV) = 250 × 250 × 126 mm^3^, number of signal averages (NSA) = 2. Figure 1b, c shows examples of treatment planning on reconstructed sagittal and coronal images.

The MR thermometry scan used was a multi-slice gradient-echo planar imaging (EPI) pulse sequence with binomial water-selective RF excitation. One stack of three coronal slices and one sagittal slice were acquired, of which the centers were aligned with the HIFU focus. A saturation slab was used to suppress signal from the water tank. The scan parameter settings were as follows: TE = 20 ms, TR = 44 ms, FA = 20°, BW = 39.5 Hz, pixel size = 2.5 × 2.5 mm^2^, slice thickness = 7 mm, FOV = 250 × 250 mm^2^, NSA = 2, EPI factor = 11, and dynamic scan duration = 3.9 s. Temperatures changes were calculated on the fly using the proton resonance frequency shift method [44, 45] and added to the baseline temperature measured with the fluoroptic probe in order to reconstruct temperature images.

The baseline *T*_1_ map, required for DCE-MRI analyses, was obtained from variable flip angle (VFA) images acquired prior to contrast agent injection. The DCE-MR images and VFA images were acquired before and after hyperthermia and were orientated parallel to the tumor-bearing leg. The VFA images were acquired with a 3D spoiled gradient-echo: TE = 1.4 ms, TR = 5 ms, FA = 5°, 10°, and 15°, BW = 192 Hz, voxel size = 1.2 × 1.5 × 2 mm^3^, FOV = 300 × 150 × 40 mm^2^, and NSA = 2. For the DCE-MR images, the same scan sequence was used with dynamic keyhole settings [46], using the last scan for the high spatial frequency data, keyhole percentage = 25 %, keyhole measurements = 2, dynamic reference scan duration = 13.1 s, dynamic keyhole scan duration = 3.3 s, total scan duration = 5 min, 47 s, and FA = 15°. MR contrast agent was injected between 15 and 20 s after starting the dynamic DCE scan. The DCE-MRI scan was acquired on average 10 min (range 6.5 to 17.5 min) after the end of hyperthermia.

For IVIM-MRI, multi-slice diffusion-weighted single-shot spin-echo EPI images were acquired before and after hyperthermia. The orientation of the slices was parallel to the tumor-bearing leg and 10 *b* values were used. The scan parameter settings were as follows: TE = 67 ms, TR = 2500 ms, FA = 90°, BW = 15.7 Hz, pixel size = 2 × 2 mm^2^, slice thickness = 3 mm, FOV = 140 × 179 mm^2^, number of slices = 12, NSA = 6, and *b* = 800, 600, 400, 200, 150, 100, 75, 50, 25, 0 s/mm^2^. A frequency-selective adiabatic inversion pulse was used for fat suppression, with a delay time of 90 ms. The IVIM-MRI scan was acquired on average 35 min (range 26 to 43 min) after the end of hyperthermia.

### MR-HIFU-induced mild hyperthermia

Mild hyperthermia (40 to 42 °C) was induced locally in all five rabbits using the clinical MR-HIFU therapy system described earlier. Sonications were performed with 60 W acoustic power at an operating frequency of 1.2 MHz, and the acoustical energy was delivered along concentric circular sub-trajectories of 4 and 8 mm diameter by electronically steering the focus, the so-called HIFU cell [47]. Mild hyperthermia was achieved by the binary feedback-loop described by Partanen et al. [48], which uses the temperature measurements provided by the MR thermometry. After initial heating to mild hyperthermic temperatures, hyperthermia was maintained by re-sonicating the sub-trajectories using a binary feedback-loop. In this study, the binary feedback-loop was slightly adapted: re-sonication was done with 80 % of the initial acoustical power instead of 50 %.

The hyperthermia protocol consisted of three hyperthermia blocks of 10 min, separated by periods of cooling. Each subsequent hyperthermia sonication started when the temperature, measured by the fluoroptic temperature probe, had decreased to the baseline temperature measured prior to heating. A hyperthermia block was considered unsuccessful and was redone after cooling down when the duration of the hyperthermia block was less than half of the intended duration, for example, due to automatic abortion by the system upon detection of large motion.

The measured hyperthermic temperatures by MR thermometry were expressed by the measures *T*_10_, *T*_50_, and *T*_90_. *T*_50_ indicates the median temperature or the temperature that was exceeded by 50 % of the target region. Similarly, *T*_10_ and *T*_90_ indicate that the temperature was exceeded by 10 and 90 % of the target region, respectively. These values were calculated over manually selected circular regions of interest (ROI) with a diameter of 10 mm at the heated area in each slice, slightly larger than the HIFU cell size. Temporal mean values were calculated for the entire hyperthermia duration, i.e., the time period between the start and end of the hyperthermia maintenance phase of the feedback algorithm of each block, for the sagittal slice and the central coronal slice.

### Tumor VOIs

For all datasets, a 3D tumor volume of interest (VOI) was selected by manual delineation of the tumor region in each slice. This tumor VOI represents the volume targeted for hyperthermia. The tumor VOIs were used for the comparison of the parameters before and after hyperthermia. In the DCE datasets, the delineation was performed after the bolus passage at the 20th dynamic; in the IVIM datasets, the delineation was performed at *b* = 0 s/mm^2^.

### DCE data analysis

The DCE-MRI analysis was performed in Matlab (2013b, Mathworks, Natick, MA). First, dynamic 3D concentration maps were reconstructed from the DCE data, using the *T*_1_ baseline maps obtained from the VFA data [49]. Second, arterial input functions (AIFs) were measured in the feeding artery of rabbit 5 in both the pre- and post-hyperthermia data. These representative concentration-time curves were parameterized using a gamma variate function [50]. The blood plasma volume fraction *v*_*p*_ has been reported to be a crucial parameter for the assessment of tumor physiological response to hyperthermia and thus should be included in the analysis [14, 15]. Therefore, the extended Tofts DCE model [18, 51] was used:

1 $$ C(t)={K}^{\mathrm{trans}}{\displaystyle \underset{0}{\overset{t}{\int }}}{C}_p\left(\tau \right)\ {e}^{k_{\mathrm{ep}}\left(t-\tau \right)}d\tau +{v}_p{C}_p(t), $$

where *K*^trans^ is the volume transfer constant between blood plasma and the extracellular extravascular space (EES), *k*_ep_ is the rate constant between the EES and the blood plasma, and *C*_*p*_(*τ*) is the concentration-time curve in the arterial blood plasma or the AIF. Equation 1 was fitted voxel-wise to the dynamic concentration maps using an iterative nonlinear least squares fit procedure, where the parameterized pre- and post-hyperthermia AIFs were used for the analysis of the pre- and post-hyperthermia data, respectively. Maps were reconstructed of *K*^trans^, *k*_ep_, and *v*_*p*_.

### IVIM data analysis

The IVIM-MRI analysis was performed using the data driven Bayesian modeling method described by Orton et al. [52], which has no user-defined parameters and is therefore robust and reproducible [52]. The method was implemented in Mathematica (7.0, Wolfram Research Inc., Champaign, IL), and the following bi-exponential model was used:

2 $$ S(b)={S}_0\left({f}_p\ {e}^{-b\cdot {D}_p}+\left(1-{f}_p\right)\ {e}^{-b\cdot {D}_t}\right), $$

where *D*_*t*_ is the true diffusion, *f*_*p*_ is the perfusion fraction, and *D*_*p*_ is the pseudo diffusion, induced by the vascular components. The Bayesian modeling method makes Gaussian approximations of the IVIM parameter histograms, resulting from least squares fitting of Eq. 2. These approximations are used as prior distributions to push outlier estimates with high uncertainty towards the center of the histogram [52]. To fill the prior distribution appropriately, the muscle surrounding the tumor was included and any water from the tank was excluded. Maps were reconstructed of *D*_*t*_, *f*_*p*_, and *D*_*p*_.

### Detection of changes after hyperthermia

While the mean of a VOI is an often used metric for the comparison of parameter values, histograms are less arbitrary and capture heterogeneity [6, 14, 37, 53]. Histograms were made for each DCE and IVIM parameter, with ranges of 0 to 5 for *K*^trans^ [min^− 1^] and *k*_ep_ [min^− 1^], 0 to 1 for *v*_*p*_ [fraction] and *f*_*p*_[fraction], 0 to 3 for *D*_*t*_ [10^− 3^mm^2^/s], and 0 to 30 for *D*_*p*_[10^− 3^mm^2^/s]. All data were distributed in 100 bins, and the bin heights were expressed in percentage of the tumor VOI volume.

For quantitative comparison, we determined the median values of all values inside the mentioned ranges (excluding the outliers). The parameter distributions were expected to be non-normal owing to tumor heterogeneity, and the pre- and post-hyperthermia data were unpaired since the tumor VOIs were delineated individually. Therefore, the Mann-Whitney *U* test was used, which was also performed in a region of interest in the surrounding muscle to test the significance of the changes in the median values. The region in the surrounding muscle used for the analyses was selected in the central slice through the tumor. The muscle region size was 10 × 10 voxels for DCE and 5 × 5 voxels for IVIM. The selected muscle region was smaller for IVIM than for DCE, because of the lower resolution and the limited availability of surrounding muscle tissue for which the IVIM parameters were extracted. Statistical tests were performed in Matlab (2013b, Mathworks, Natick, MA), and a *p* value of less than 0.001 was considered indicative of a statistically significant difference.

Two-dimensional cross-correlation histograms provide insight in the inter-relationships between parameters [34] and were made for the following combinations: *v*_*p*_ × *K*^trans^, *k*_ep_ × *K*^trans^, *v*_*p*_ × *k*_ep_, *f*_*p*_ × *D*_*t*_, *D*_*p*_ × *D*_*t*_, and *f*_*p*_ × *D*_*p*_. The same number of bins and ranges were used as for the individual parameter histograms, and the intensities were expressed in percentage of the tumor VOI volume.

To ensure that observed changes in parameter values were induced by hyperthermia, data reproducibility was tested. The IVIM scan of rabbit 1 after hyperthermia was repeated, and the results were compared. Since the DCE scans require the use of a contrast agent, a similar reproducibility test was deemed not feasible for DCE-MRI.

## Results

The T2w MR images acquired during the planning phase showed that all rabbits had one tumor except for rabbit 4, which had three small contiguous tumors. Rabbit 2 had a large necrotic core in the tumor and died during the last few minutes of the hyperthermia treatment.

### Tumor VOIs

In the MR images, it could be observed that all rabbits had one tumor except for rabbit 4, which had three small contiguous tumors. In rabbit 2, a large necrotic core was observed. Table 1 shows the volumes of the tumor VOIs, for which the DCE and IVIM analyses were performed. The discrepancy between the volumes delineated in the DCE and in the IVIM data can be attributed to the differences in the MR images (voxel size, contrast, geometrical distortions by EPI). Note that the volumes before and after hyperthermia were comparable.

**Table 1:** Tumor VOI volume (cm^3^)

Rabbit number	DCE		IVIM	
	Pre	Post	Pre	Post
	HT	HT	HT	HT
1	13	13	19	19
2	13	13	20	20
3	12	12	17	18
4	7	7	13	13
5	10	11	17	19

*HT* hyperthermia

### MR-HIFU-induced mild hyperthermia

**Fig. 2:**
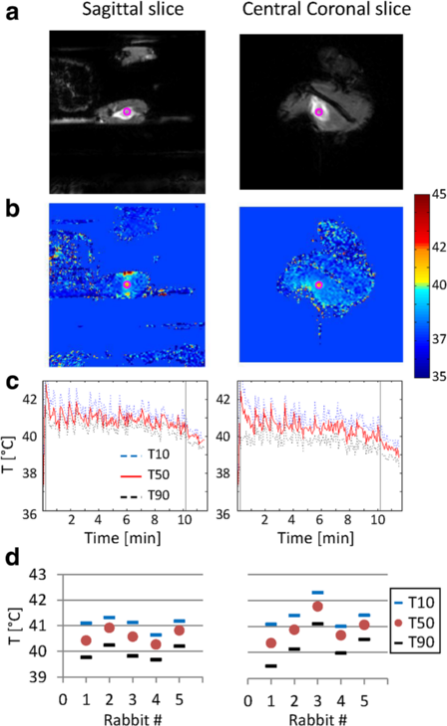
MR-HIFU-induced hyperthermia. Examples of magnitude images (**a**) and temperature images (**b**) of the MR thermometry sequence and *T*_10_, *T*_50_, and *T*_90_ profiles over time (**c**) (rabbit 2, first hyperthermia block). In (**c**), the *vertical lines* indicate the start and end of the hyperthermia maintenance phase of the feedback algorithm. The ROIs used for the *T*_10_, *T*_50_, and *T*_90_ calculation are indicated by the *pink circles* in the images in (**a**, **b**). In (**d**), the temporal mean values of *T*_10_, *T*_50_, and *T*_90_ over the entire hyperthermia duration are shown per rabbit

Three 10-min blocks of mild hyperthermia (40 to 42 °C) were successfully achieved in all five rabbits using MR-HIFU. In rabbit 2, one mild hyperthermia block was manually aborted because of observed motion artifacts in the MR thermometry; in rabbit 4, one mild hyperthermia block was automatically aborted because of connection loss between the MR console and the HIFU console. More details on the mild hyperthermia durations are given in Table 2. Figure 2a, b shows examples of magnitude and temperature images of the MR thermometry sequence; examples of the temporal profiles of *T*_10_, *T*_50_, and *T*_90_ of the corresponding ROIs (circles) are shown in Fig. 2c. The mean values of *T*_10_, *T*_50_, and *T*_90_ over the entire hyperthermia duration are shown in Table 3 and plotted in Fig. 2d for each rabbit. All *T*_50_ values were within the desired hyperthermic temperature range of 40 to 42 °C. In the coronal slice of rabbit 3, the mean *T*_10_ was higher than 42 °C and the mean *T*_50_ was higher than the other rabbits.

**Table 2:** Details on MR-HIFU mild hyperthermia of rabbit Vx2 tumors

Block	Rabbit 1	Rabbit 2	Rabbit 3	Rabbit 4	Rabbit 5
HT	8 min	10 min	4 min	135 s^b^	10 min
Cool	5 min	5 min	8 min	13 min	11 min
HT	10 min	70 s^a^	10 min	10 min	10 min
Cool	5 min	3 min	9 min	5 min	17 min
HT	10 min	10 min	10 min	10 min	10 min
Cool		5 min		5 min	
HT		10 min		10 min	
Total HT duration	28 min	31 min	24 min	32 min	30 min

*HT* hyperthermia

^a^Manually aborted: motion artifacts in the MR thermometry observed

^b^Automatically aborted: connection loss between the MR console and the HIFU console

**Table 3:** Temporal mean and standard deviation (SD) of *T*_50_, *T*_90_, and *T*_10_, averaged over the three hyperthermia blocks

Rabbit	Central coronal slice			Sagittal slice		
	*T*_50_ (°C)	*T*_90_ (°C)	*T*_10_ (°C)	*T*_50_ (°C)	*T*_90_ (°C)	*T*_10_ (°C)
1	40.4	39.5	41.1	40.4	39.8	41.1
2	40.9	40.1	41.4	40.9	40.2	41.3
3	41.8	41.1	42.3	40.6	39.8	41.1
4	40.7	40.0	41.0	40.3	39.7	40.6
5	41.1	40.5	41.4	40.8	40.2	41.2
Mean (SD)	40.9 (0.5)	40.2 (0.6)	41.4 (0.5)	40.6 (0.3)	39.9 (0.3)	41.1 (0.3)

### Reproducibility

**Fig. 3:**
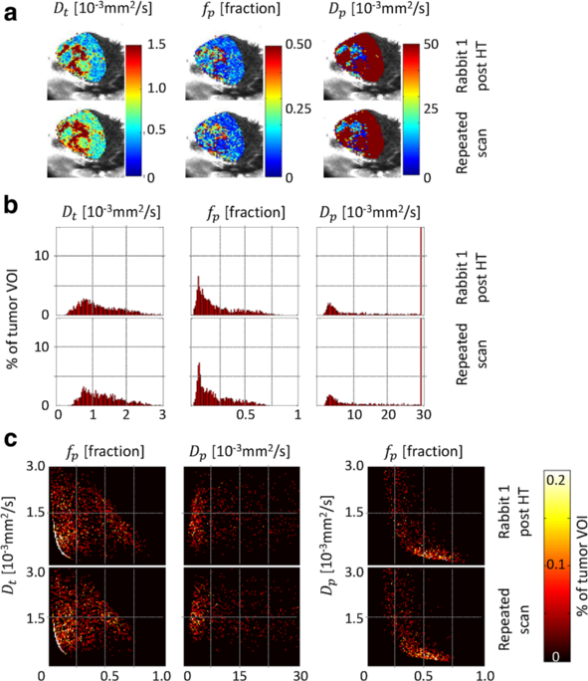
Reproducibility results. Results of the repeated IVIM scans acquired in rabbit 1 after hyperthermia. The parameter maps of the central slice through the tumor are shown in (**a**), the individual parameter histograms in (**b**), and the two-dimensional cross-correlation histograms in (**c**). The parameter maps in frame (**a**) are shown as an overlay over the corresponding magnitude image (*b* = 0 s/mm^2^)

In Fig. 3, the results of the repeated IVIM scans of rabbit 1, acquired post-hyperthermia, are shown. The parameter maps of the central slice through the tumor (Fig. 3a) look similar, as well as the individual parameter histograms (Fig. 3b) and the cross-correlation histograms (Fig. 3c), except for some minor differences in *D*_*t*_. Table 4 shows that the median *D*_*t*_ values showed a small but statistically significant difference of 0.14 × 10^−3^ mm^2^/s (*p* < 0.001), while no significant difference was found between the median *f*_*p*_ (*p* = 0.14) and *D*_*p*_ values (*p* = 0.09).

**Table 4:** Reproducibility test: median IVIM parameter values of two dynamic scans acquired in rabbit 1, post-hyperthermia (HT)

	Rabbit 1 post-HT	Repeated scan	*p* value
*D*_*t*_ [10^−3^ mm^2^/s]	1.06	1.20	*<0.001*
*f*_*p*_ [fraction]	0.16	0.17	*0.14*
*D*_*p*_ [10^−3^ mm^2^/s]	5.2	6.1	*0.09*

### DCE and IVIM parameter maps

**Fig. 4:**
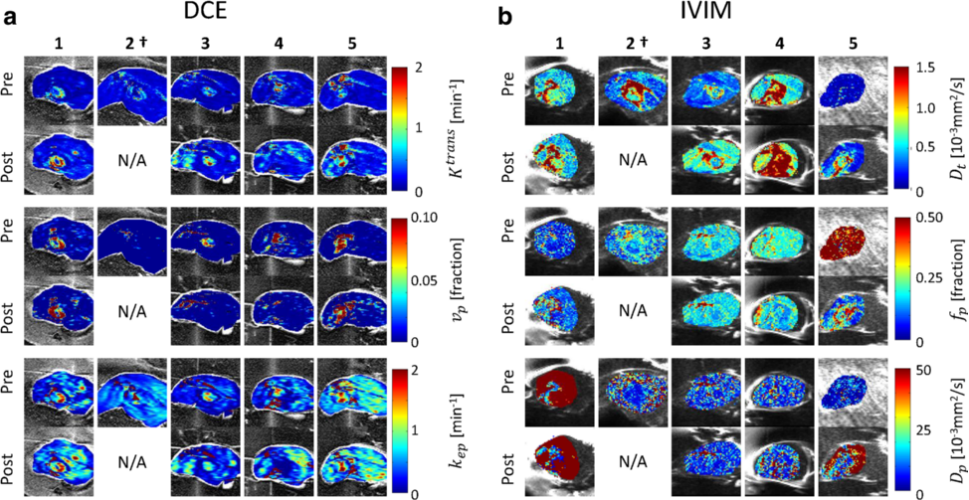
DCE and IVIM parameter maps. Pre- and post-hyperthermia DCE (**a**) and IVIM (**b**) parameter maps of the central slices through the tumor for all rabbits (1-5). The parameter maps are shown as an overlay over the corresponding magnitude image (20^th^ dynamic for DCE and *b* = 0 s/mm^2^ for IVIM). Rabbit 2 (†) died during the treatment.

The DCE and IVIM parameter maps of the central slice through each tumor are displayed in Fig. 4. Please note that the region for which the IVIM analysis was performed is limited to the region included for the Bayesian prior distribution, for which any water from the tank was avoided in the delineation. Variations in the parameter maps can be observed between the rabbits, both before and after hyperthermia. Rabbit 2 died during treatment; the corresponding data were excluded from the analysis. The signal-to-noise ratio of the pre-hyperthermia IVIM data acquired in rabbit 5 was very low. We therefore decided to refrain from including IVIM data from this animal in any comparisons. Decreased values can be observed in the post-hyperthermia *v*_*p*_ map of rabbit 3 (*v*_*p*_ < 0.02) and in all post-hyperthermia DCE maps of rabbit 4 (*v*_*p*_ < 0.02, *K*^trans^ < 0.4, and *k*_ep_ < 0.4; Fig. 4a).

### Histograms and median values

**Fig. 5:**
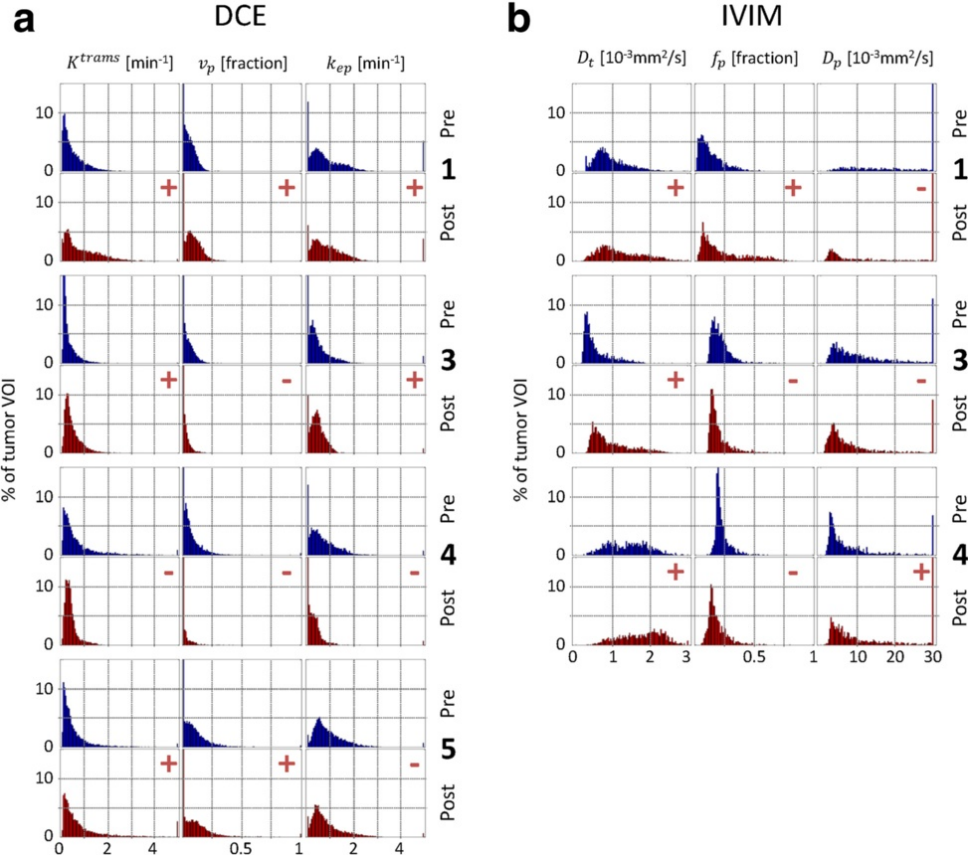
DCE and IVIM parameter histograms. Histograms of the pre- and post-hyperthermia DCE parameters (**a**) and of the IVIM parameters (**b**) in the tumor VOI of the rabbits included in the analysis. Median changes are indicated at the *top right* of each post-hyperthermia histogram: *+* increase, − decrease

Pre- and post-hyperthermia histograms of the DCE parameters in the tumor VOIs are shown in Fig. 5a. Variations in the pre-hyperthermia histograms can be observed between the rabbits, in particular, the *k*_ep_ histograms. Table 5 shows the comparisons of pre- and post-hyperthermia median values of the DCE parameters and the corresponding *p* values, for both the surrounding muscle and the tumor VOI. In the surrounding muscle, no significant changes were found in the median values of *v*_*p*_ in all rabbits and of *k*_ep_ in rabbits 1 and 4, while significant increases (*p* < 0.001) were found in the median values of *k*_ep_ in rabbits 3 and 5 and of *K*^trans^ in all rabbits, in the order of 0.1 min^−1^. The changes in the tumor VOI are most obvious in rabbit 4, where all three DCE parameter histograms, as well as the median values, shifted towards lower values. For all other rabbits, the *K*^trans^ histograms and the median *K*^trans^ values shifted towards higher values and the changes in median values were in the order of 0.2 min^−1^. The median *v*_*p*_ increased in rabbits 1 and 5 and decreased in rabbits 3 and 4. In the *k*_ep_ histograms of rabbits 3 and 4, clear shape changes can be observed. The median *k*_ep_ increased in rabbit 1 and decreased in rabbits 4 and 5. In rabbit 3, the bulk shift in the *k*_ep_ distribution towards lower values changed the skewness (Fig. 5a). This resulted in an increase in the median *k*_ep_ (Table 5), which does not reflect the observed changes in the histogram (*p* = 0.006).

**Table 5:** Median DCE parameter values in the surrounding muscle and in the tumor VOI, pre- and post-hyperthermia

Surrounding muscle	*K* ^trans^ [min^−1^]				*v*_*p*_ [fraction]				*k*_ep_ [min^−1^]			
Rabbit	Pre	Post		*p*	Pre	Post		*p*	Pre	Post		*p*
1	0.14	0.30	+	<0.001	0.003	0.001		0.077	0.65	0.74		0.004
3	0.08	0.20	+	<0.001	0.000	0.000		0.021	0.26	0.37	+	<0.001
4	0.16	0.26	+	<0.001	0.000	0.000		0.476	0.43	0.48		0.247
5	0.13	0.27	+	<0.001	0.000	0.000		0.817	0.81	0.96	+	<0.001
Tumor VOI	*K* ^trans^ [min^−1^]				*v*_*p*_ [fraction]				*k*_ep_ [min^−1^]			
Rabbit	Pre	Post		*p*	Pre	Post		*p*	Pre	Post		*p*
1	0.34	0.68	+	<0.001	0.055	0.085	+	<0.001	0.68	0.80	+	<0.001
3	0.14	0.34	+	<0.001	0.043	0.024	−	<0.001	0.36	0.41		0.006
4	0.39	0.29	−	<0.001	0.053	0.030	−	<0.001	0.57	0.26	−	<0.001
5	0.30	0.47	+	<0.001	0.087	0.110	+	<0.001	0.74	0.62	−	<0.001

Plus signs (+) indicate significant increase after hyperthermia, and minus signs (−) indicate significant decrease after hyperthermia

Pre- and post-hyperthermia histograms of the IVIM parameters in the tumor VOIs are shown in Fig. 5b. It is notable that all pre-hyperthermia histograms look different in shape. Table 6 shows the comparisons of the pre- and post-hyperthermia median values of the IVIM parameters and the corresponding *p* values, for both the surrounding muscle and the tumor VOI. In the surrounding muscle, no significant changes were found in the median values of *f*_*p*_ in all rabbits and of *D*_*t*_ in rabbit 1 and of *D*_*p*_ in rabbits 3 and 4. The significant changes in *D*_*t*_ in rabbits 3 and 4 were in the order of 0.2 × 10^−3^ mm^2^/s and in *D*_*p*_ in rabbit 1 was 700 × 10^−3^ mm^2^/s. For the tumor VOI, all *D*_*t*_ histograms shifted towards higher values after hyperthermia and the changes in the median values in rabbits 3 and 4 were in the order of 0.4 × 10^−3^ mm^2^/s. In the histograms and median values of *f*_*p*_ an increase was found in rabbit 1 and a decrease in rabbits 3 and 4. The pre-hyperthermia *D*_*p*_ histogram of rabbit 1 shows an even distribution covering a wide range, and the median value was much larger than all other median *D*_*p*_ values (Table 6). The median *D*_*p*_ value decreased in rabbit 3 and increased in rabbit 4.

**Table 6:** Median IVIM parameter values in surrounding muscle and in the tumor VOI, pre- and post-hyperthermia

Surrounding muscle	*D*_*t*_ [10^−3^ mm^2^/s]				*f*_*p*_ [fraction]				*D*_*p*_ [10^−3^ mm^2^/s]			
Rabbit	Pre	Post		*p*	Pre	Post		*p*	Pre	Post		*p*
1	0.42	0.46		0.670	0.07	0.10		0.026	300	1000	+	<0.001
3	0.32	0.60	+	<0.001	0.16	0.18		0.642	5.6	5.4		0.509
4	0.46	0.55	−	<0.001	0.18	0.16		0.060	6.6	8.2		0.497
Tumor VOI	*D*_*t*_ [10^−3^ mm^2^/s]				*f*_*p*_ [fraction]				*D*_*p*_ [10^−3^ mm^2^/s]			
Rabbit	Pre	Post		*p*	Pre	Post		*p*	Pre	Post		*p*
1	0.82	1.06	+	<0.001	0.10	0.16	+	<0.001	13.5	5.2	−	<0.001
3	0.40	0.76	+	<0.001	0.19	0.17	−	<0.001	7.6	5.0	−	<0.001
4	1.40	1.82	+	<0.001	0.20	0.16	−	<0.001	4.7	6.0	+	<0.001

Plus signs (+) indicate significant increase after hyperthermia, and minus signs (−) indicate significant decrease after hyperthermia

### Cross-correlation histograms

**Fig. 6:**
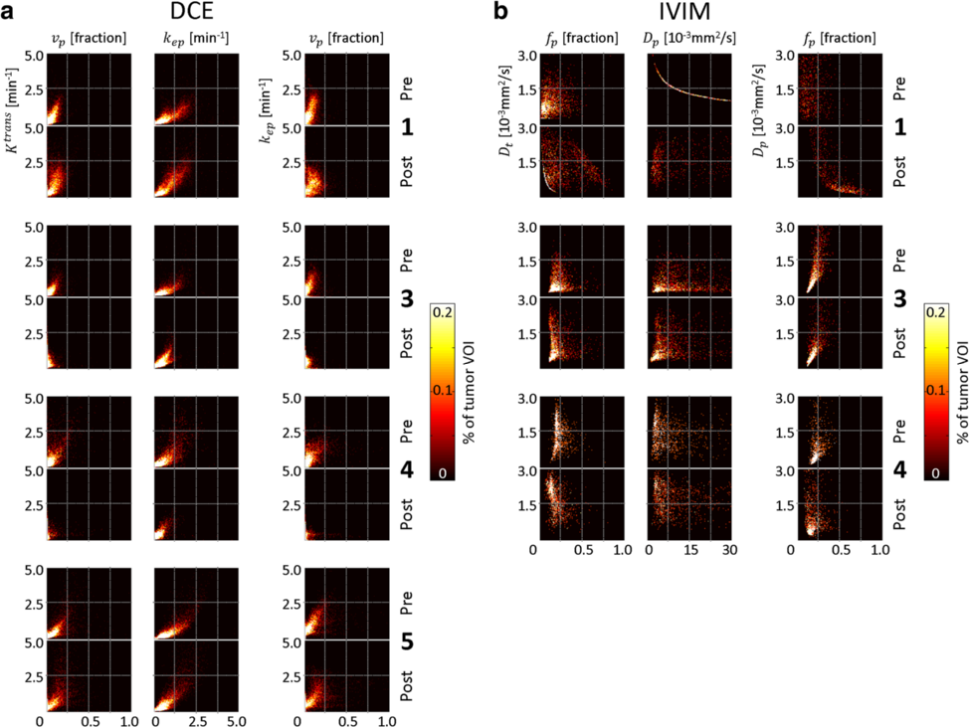
Cross-correlation histograms of DCE and IVIM parameters. Cross-correlation histograms of the pre- and post-hyperthermia DCE parameters (**a**) and of the IVIM parameters (**b**) in the tumor VOIs of the rabbits included in the analysis

The two-dimensional cross-correlation histograms of the DCE parameters in the tumor VOIs are displayed in Fig. 6a. The shapes of the different pre-hyperthermia cross-correlation histograms are comparable for rabbits 1, 3, and 5; for rabbit 4, the shapes are less elongated and more diffuse. After hyperthermia, the cross-correlation histograms of rabbits 1 and 5 become more diffuse, while those of rabbits 3 and 4 become more compact, particularly the *v*_*p*_ × *K*^trans^ and *v*_*p*_ × *k*_ep_ histograms.

The cross-correlation histograms of the IVIM parameters in the tumor VOIs are displayed in Fig. 6b. In rabbit 1, the pre-hyperthermia *D*_*p*_ × *D*_*t*_ histogram shows a strong correlation between the parameters for all voxels, which indicate systematic errors in the parameter estimation. Similarly, the post-hyperthermia *f*_*p*_ × *D*_*t*_ histogram of rabbit 1 shows a strong correlation between the parameters for a large portion of the voxels. The cross-correlation histograms of rabbits 3 and 4 have similar shapes but differ in their distributions: in rabbit 4, there were more voxels with low *f*_*p*_ values in combination with high *D*_*t*_ values.

## Discussion

DCE-MRI and IVIM-MRI data were acquired before and after MR-HIFU-induced hyperthermia in rabbits with Vx2 tumors. The pre-hyperthermia DCE and IVIM parameter maps and histograms revealed variations between the rabbits. This implies that the group was heterogeneous in terms of DCE and IVIM parameter distributions. This heterogeneity was also observed in five other rabbits that did not receive the hyperthermia treatment and were therefore not included in this study (data not shown). The post-hyperthermia data were acquired within 1 h after hyperthermia. Although the duration of the physiological effects after applying hyperthermia is a controversial aspect [54], several studies have shown that changes in regional blood flow and permeability persisted and could be detected up to a few hours after hyperthermia [10, 12, 13]. Therefore, it is assumed that the effect of differences in timing of the image acquisitions on the parameter changes is limited, as compared to other sources. The changes in the DCE and IVIM parameters after hyperthermia varied between the rabbits and are discussed below.

Rabbit 4 had three contiguous small tumors instead of a single tumor and received a few minutes longer hyperthermia than the other rabbits. This combination may have led to a different physiological response in this rabbit as compared to the other rabbits. In the other rabbits (rabbits 1, 3, and 5), increases in *K*^trans^ were observed after hyperthermia which were a factor 2 larger than the changes observed in the surrounding muscle. For the median *k*_ep_ values, only the change in rabbit 4 could be considered to be significant; the changes in the other rabbits were in the same order as those found in the surrounding muscle. The changes in the median *K*^trans^ and *k*_ep_ values were in the order of 0.2 min^−1^, where extreme outliers were excluded to have a realistic representation of the parameter value distribution. Hijnen et al. [38] showed *K*^trans^ changes of about 0.1 min^−1^ after hyperthermia in tumor-bearing mice in the non-necrotic tumor areas, which is in the same order of magnitude as the changes observed in this study. In the whole tumor, changes in the mean *K*^trans^ and *k*_ep_ values were smaller (0.017 and 0.022 min^−1^). Discrepancies between the results may be explained by the different tumor and animal models. In addition, a different model was used for the DCE analysis, standard Tofts [55] versus extended Tofts [18], resulting in a discrepancy in the permeability and flow contributions in *K*^trans^ [56].

The changes in *v*_*p*_ with (*p* < 0.001) in the tumor VOI can be considered to be significant, as no significant changes were observed in the surrounding muscle. An increase in *v*_*p*_ was observed in rabbits 1 and 5 and a decrease in rabbit 3. Interestingly, hyperthermic temperatures measured in the coronal slice of rabbit 3 were higher than in the other rabbits: the mean *T*_50_ was close to 42 °C and the mean *T*_10_ was higher than 42 °C. Since *v*_*p*_ represents the blood plasma volume fraction in a voxel, it is strongly related to the size of the vessels. It is well known that tumor capillaries are hastily formed and lack the ability to actively dilate. However, tumor capillaries may passively dilate upon hyperthermia, as a result of increased blood flow in adjacent tissue or of increased cardiac output [7], which may explain the increase in *v*_*p*_ observed in rabbits 1 and 5. At moderate hyperthermic temperatures (>42 °C), reduced tumor vessel diameters have been reported in Vx2 tumors in rabbit ear chambers [8], which may explain the observed *v*_*p*_ decrease in rabbit 3. Dudar and Jain [8] suggested that the reduction of tumor vessel diameters may be attributed to swelling of the endothelial cells and tissue parenchyma, induced by a decreased pH in tumors during hyperthermia. The *k*_ep_ histogram shapes of rabbits 3 and 4 clearly changed after hyperthermia to a more compact distribution. It is notable that these are the same rabbits that showed a decrease in *v*_*p*_. This potential relation can be seen more clearly in the *v*_*p*_ × *k*_ep_ histograms of these rabbits.

For the IVIM analysis, the reproducibility was tested by comparing a repeated IVIM scan. No significant differences were found between the *f*_*p*_ and *D*_*p*_ histograms and median values (*p* = 0.14 and *p* = 0.09); the changes in *D*_*t*_ were significantly different and relatively small compared to the median values (13 and 12 %).

The pre-hyperthermia IVIM data of rabbit 5 had a low signal-to-noise ratio, possibly due to motion during the acquisition; hence, a reliable comparison with the post-hyperthermia data was not possible. A clear increase in *D*_*t*_ after hyperthermia could be seen in rabbits 1, 3, and 4. The changes in the median values were factors of 1.7, 2.6, and 3.0 times larger than the changes found in the reproducibility test and factors of 6, 1.3, and 4.7 times larger than the changes found in the surrounding muscle. This indicates that the observed changes in *D*_*t*_ are likely to be induced by hyperthermia. For *f*_*p*_, no significant changes were found in the surrounding muscle. In the tumor VOI, an increase in *f*_*p*_ was observed in rabbit 1 and a decrease in rabbits 3 and 4, similar to the changes observed in *v*_*p*_. While the interpretations of *f*_*p*_ and *v*_*p*_ are different, signal fraction and volume fraction, respectively, the parameters are strongly related to each other as they both reflect the intra-voxel fraction of the vascular component.

The *f*_*p*_ values in rabbit 1 are much lower than in the other rabbits, which indicate an overall small contribution of vascular components to the signal. The cross-correlation histograms of these data revealed a strong correlation between *D*_*t*_ and *D*_*p*_, suggesting systematic errors in the parameter estimation. The low *f*_*p*_ values are likely the reason for the systematic *D*_*p*_ estimation errors, inasmuch *D*_*p*_ cannot be estimated accurately when *f*_*p*_ is too low [57, 58].

While the results show that changes in DCE and IVIM parameters after MR-HIFU-induced hyperthermia could be detected, the changes were found to be variable between the rabbits. The group appeared to be heterogeneous in terms of DCE and IVIM parameter distributions, and it is likely that such a start condition would result in a heterogeneous outcome. In future research, stratification of starting conditions would be desirable, which requires a larger number of subjects.

## Conclusions

In this study, we have shown that DCE and IVIM parameters maps and (cross-correlation) histograms could be constructed to detect changes after MR-HIFU-induced hyperthermia in rabbit Vx2 tumors. Perfusion parameter histograms provided insight into the changes of the parameter distributions and showed that changes in most of the median values were statistically significant (*p* < 0.001). However, the detected changes were variable between the rabbits. The results suggest that DCE- and IVIM-MRI may be promising tools to assess tumor physiology responses to hyperthermia. Further research in a larger number of subjects is necessary to assess their value for treatment response monitoring.
